# Fosfomycin and Its Derivatives: New Scale Inhibitors for Oilfield Applications

**DOI:** 10.1021/acsomega.2c00429

**Published:** 2022-03-15

**Authors:** Mohamed F. Mady, Rocio Ortega

**Affiliations:** † Department of Chemistry, Bioscience and Environmental Engineering, Faculty of Science and Technology, University of Stavanger, N-4036 Stavanger, Norway; ‡ Department of Green Chemistry, National Research Centre, Dokki, Cairo 12622, Egypt

## Abstract

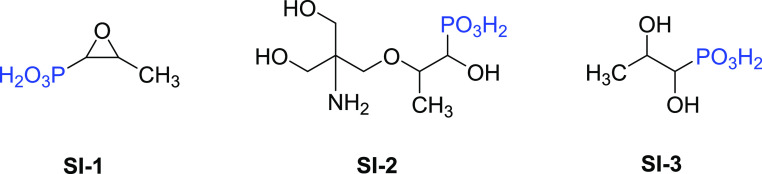

Aminomethylenephosphonate-based scale inhibitors (SIs) have been widely studied and recognized for several decades to mitigate various oilfield scales. However, most of these compounds afforded several drawbacks, such as poor biodegradability and intolerance with the production system. As environmental regulations become more rigid, new production chemicals must adhere to certain criteria to qualify for use in the oil and gas industry, particularly in areas with strict regulations, such as the Norwegian Sea. The low toxicity of fosfomycin encouraged us to test fosfomycin and related molecules as new aminomethylene-free phosphonate SIs for calcite and gypsum scales. The tested chemicals are fosfomycin disodium salt (**SI-1**), fosfomycin trometamol (**SI-2**), and hydrolysis of fosfomycin called 1,2-dihydroxypropyl phosphonic acid (**SI-3**). The inhibition efficiency of all these chemicals was evaluated against calcite and gypsum scales compared to commercial oilfield scale inhibitor hydroxyphosphonoacetic acid (**HPAA**) according to the NACE Standard TM0374-2007. In addition, the calcite scale inhibition efficiency of all aminomethylene-free phosphonate SIs (**SI-1** to **SI-3** and **HPAA**) was investigated based on the Heidrun oilfield, Norway. Moreover, we have reported the calcium compatibility of these chemicals at various concentrations of SIs and calcium ions at 80 °C over 24 h. All new aminomethylene-free phosphonate SIs showed good gypsum and calcite inhibition performance. It was also found that all tested chemicals derived from fosfomycin demonstrated excellent compatibility with calcium ions of up to 1000 ppm throughout the 24 h experiment period compared to **HPAA**.

## Introduction

Scale formation leads to critical flow assurance problems in oil and gas production installations.^1^ It refers to the precipitation of inorganic minerals due to favored supersaturation conditions found in formation waters. The life cycle of the petroleum reservoir plays a vital role in the scale formation process.^2^ Scale formation is more common in mature oilfields, where seawater is reinjected into the reservoir to enhance oil recovery (EOR). Scale can be precipitated over time, eventually hindering the production process, triggering a chain of problems, thereby decreasing oilfield productivity, causing economic and potential loss of the well. So, there is a clear need in preventing the formation of scale precipitates.^3,4^

Calcite (CaCO_3_), barite (BaSO_4_), gypsum (CaSO_4_·2H_2_O), and celestite (SrSO_4_) are among the most common divalent metal ion precipitates encountered in the oilfield industry. Calcite is the most commonly encountered scale deposit and the most thermodynamically stable polymorph of calcium carbonate.^5^ Water found within carbonate and calcite-cemented sandstone petroleum reservoirs usually contains high concentrations of Ca^2+^ and Mg^2+^ ions.^6,7^ Furthermore, seawater carries high concentrations of sulfate (SO_4_^2–^) ions. The difference in the ionic nature of seawater and formation water when mixed during EOR leads to scale formation. Sulfate scales are more commonly formed when formation water and injected seawater are mixed or when two different waters mix in topside flowlines. For example, the gypsum scale is commonly found in heat exchangers and oilfield applications.^8^

One of the most commonly used techniques to overcome scaling problems is the deployment of scale inhibitors (SIs).^9^ SIs are usually hydrophilic chemicals utilized to inhibit nucleation and/or retard crystal growth of inorganic scales.^10^ Depending on their chemical structure, SIs present different features that make them unique for certain applications and ensure an unhindered flow of hydrocarbons through production pipelines. Polyphosphates, phosphate esters, non-polymeric phosphonates, aminophosphonates, polyphosphonates, polysulfonates, and polycarboxylates are among the most common classes of SIs used in oilfield applications.^11^ Most current SIs have a trade-off between inhibition performance and costs. However, they lack other important characteristics that must be considered before their application in the field, such as calcium tolerance and biodegradability.^12^ In addition, it is required that remarkable SIs present high thermal stability for squeeze treatment applications.

**Figure 1:**
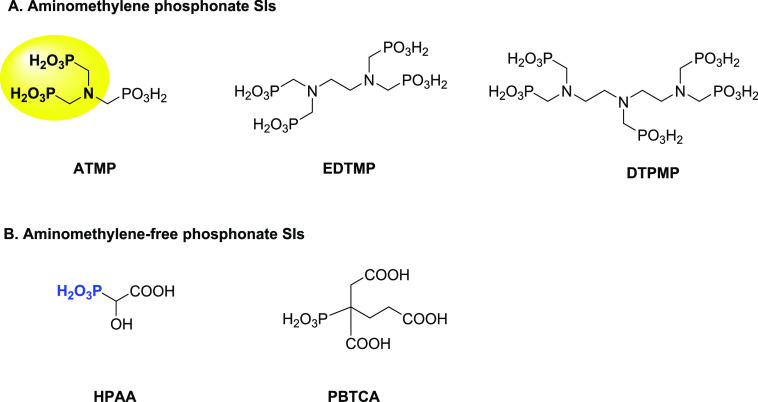
Schematic representation of chemical structures of a series of aminomethylene phosphonate SIs (A: **ATMP**, **EDTMP**, and **DTPMP**) and aminomethylene-free phosphonate SIs (B: **HPAA** and **PBTCA**) in the upstream oil and gas industry.

Aminomethylenephosphonate compounds have been widely studied and recognized as commercial SIs, notably for squeeze treatment. These chemicals showed excellent adsorption activity onto the formation rock, providing long squeeze lifetimes. However, a drawback is that classic SIs such as aminotrismethylenephosphonic acid (**ATMP**), diethylenetriaminepentamethylene phosphonic acid (**DTPMP**), and ethylenediamine tetramethylene phosphonic acid (**EDTMP**) demonstrate low biodegradability, implying that they cannot be used in offshore regions with strict environmental regulations, such as the North Sea (Figure 1). Furthermore, many of these classes of SIs lack good tolerance properties with high calcium brines, leading to precipitation and deposition of a Ca^2+^-SI complex.^13^

Aminomethylene-free phosphonates are also widely used as SIs for upstream petroleum industry applications. In an earlier study, we have tested a series of aminomethylene-free phosphonate compounds as SIs for various oilfield scales (calcite and barite) based on the Heidrun oilfield, Norwegian Sea, Norway.^14^ It was found that hydroxyphosphonoacetic acid (**HPAA**) showed outstanding inhibition performance for calcite scale and a good efficiency against oilfield barite scaling using a high-pressure dynamic tube-blocking rig at 100 °C and 1200 psi (Figure 1). However, **HPAA** exhibited moderate biodegradation properties over 28 days.^14,15^

As environmental regulations become more rigid, new production chemicals must adhere to certain criteria to qualify for use in the petroleum industry. Therefore, there is a great need to develop suitable chemicals that allow a high scale inhibition efficiency and an improved environmental footprint to fulfill the requirements set by various stakeholders.^16^ Furthermore, the drawbacks of commercial aminomethylenephosphonate SIs motivated us to synthesize and evaluate new SIs based on the aminomethylene-free phosphonate group.^11^ This study aims to synthesize aminomethylene-free phosphonates derived from fosfomycin as the starting compound and evaluate their performance as oilfield SIs. Fosfomycin belongs to the class of phosphonic antibiotics used for the treatment of urinary tract infections (UTIs).^17^ As far as our research is concerned, and to the best of our knowledge, this chemical has not been reported in the open literature for oilfield scale inhibition applications. The initiative of using fosfomycin as the starting compound rises from its low toxicity, good bioavailability, and the presence of functional groups in its structure backbone that are known to be favorable features in scale inhibitors. This set of characteristics could provide good inhibition efficiency and improved biodegradation performance in contrast to commercial SIs. Similarly, our research group has developed a series of low toxicity bisphosphonates, commonly used as bone-targeting drugs, as new SIs against carbonate and sulfate oilfield scales.^18^

**Figure 2:**
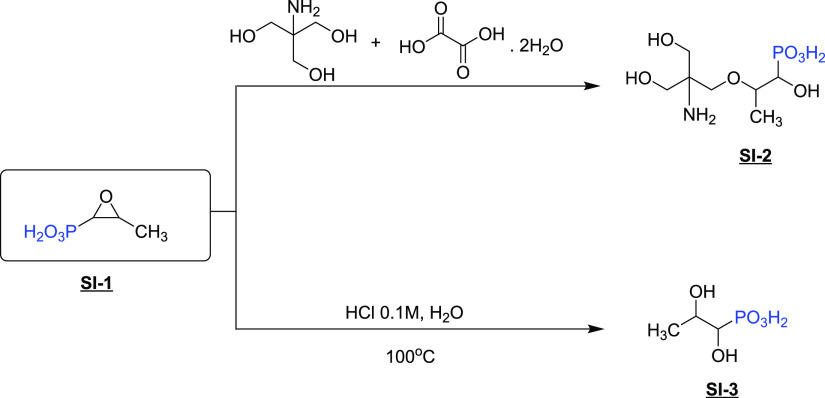
Synthesis of oilfield scale inhibitors derived from fosfomycin.

In this project, fosfomycin disodium salt (**SI-1**) was evaluated as a scale inhibitor against gypsum and calcite scales. In addition, the starting material (**SI-1**) will be used to synthesize the bioavailable version of fosfomycin, commercially known as fosfomycin trometamol (**SI-2**) (Figure 2).^19^ This structure contains an ether linkage. It has been shown that the presence of this functional group improves biodegradation performance.^20^ Another non-polymeric phosphonate-based SI was synthesized via hydrolysis of fosfomycin, affording **SI-3** (Figure 2). The inhibition performance will be assessed against calcite and gypsum scales according to the NACE Standard TM0374-2007.^21^ An additional brine composition for calcite formation simulated based on the Heidrun oilfield, Norway, was also evaluated.

## Experimental Section

### Materials and Characterization

Fosfomycin was purchased from Tokyo Chemical Industry Co., Ltd. In addition, all other chemicals used in this project were purchased from Tokyo Chemical Industry Co., Ltd., Sigma-Aldrich (Merck), VWR chemicals, and ACROS Organics. All solvents were used as purchased without further purification. Hydroxyphosphonoacetic acid (**HPAA**) was supplied from ShanDong XinTai Water Treatment Technology Co., Ltd., China. The structures of the synthesized products were characterized using nuclear magnetic resonance (NMR) spectroscopy and Fourier-transform infrared (FTIR) spectroscopy. ^1^H and ^31^P NMR chemical shifts were obtained in deuterium oxide (D_2_O) using a 400 MHz Bruker NMR spectrometer. The data was processed using TopSpinTM 3.2 software. Additionally, an Agilent Cary 630 FTIR spectrometer equipped with a diamond composite ATR (attenuated total reflectance) crystal was used. FTIR data was processed using MicroLab PC software.

### Synthesis of Fosfomycin Derivatives as New Oilfield SIs

**SI-1** was purchased from Tokyo Chemical Industry Co., Ltd. (Figure 2).

(3-Methyloxiran-2-yl)phosphonic acid (**SI-1**): IR ν_max_ (cm^–1^): 1089 (PO_3_). ^1^H NMR (D_2_O, 400 MHz) δ ppm: 3.20–3.13 (m, 1H, −C**H**–CH_3_), 2.75–2.69 (dd, 1H, −C**H**–PO_3_H_2_), 1.38–1.37 (d, 3H, −CH–C**H**_3_). ^31^P NMR (D_2_O, 162.00 MHz) δ ppm: 10.00.

#### (2-(2-Amino-3-hydroxy-2-(hydroxymethyl)propoxy)-1-hydroxypropyl)phosphonic Acid (**SI-2**)^22^

**SI-2** was prepared as described by Casazza.^22^ A 100 mL two-neck flask equipped with a reflux condenser at 65 °C and magnetic stirring was loaded with fosfomycin disodium salt (2.00 g, 10.98 mmol) in 16 mL of methanol. Another 100 mL one-neck flask with the same arrangement was set up at 50 °C and loaded with oxalic acid dihydrate (1.39 g, 10.99 mmol), tromethamine (1.33 g, 10.99 mmol), and 9 mL of methanol. After complete dissolution, the temperature of the second reaction was raised to 65 °C. After that, this solution was gradually added to the first reaction at the same temperature using a dropping funnel. After completion, the final solution was left to cool down to room temperature for 3 h and further cooled in a fridge at 4 °C overnight. The next day, the milky suspension was filtered under vacuum using a Büchner funnel. The filtrate was concentrated using a rotary evaporator. The product was further washed with 15 mL of a 1:1 mixture of acetone-ethanol under vigorous stirring at room temperature for 3 h. A suspension of white crystals was obtained and filtered under vacuum using a Büchner funnel. The obtained crystals were washed with absolute ethanol to afford a white powder (**SI-2**). The synthesis route of this reaction is shown in Figure 2.

(2-(2-Amino-3-hydroxy-2-(hydroxymethyl)propoxy)-1-hydroxypropyl)phosphonic acid (**SI-2**): Yield: 86%. IR ν_max_ (cm^–1^): 3048 (NH_2_), 2945, 2822 (OH), 1138 (CO), 1035 (PO_3_). ^1^H NMR (D_2_O, 400 MHz) δ ppm: 3.60 (s, 6H, (C**H**_2_(OH))_2_–C(NH_2_)–C**H**_2_−), 3.27–3.20 (m, 1H, −C**H**–CH_3_), 2.87–2.80 (dd, 1H, −C**H**(OH)(PO_3_H_2_)), 1.37–1.35 (d, 3H, −CH–C**H**_3_). ^31^P NMR (D_2_O, 162.00 MHz) δ ppm: 12.29.

#### 1,2-Dihydroxypropyl Phosphonic Acid (**SI-3**)

A 50 mL one-neck flask equipped with a reflux condenser at 100 °C and magnetic stirring was loaded with fosfomycin disodium salt (2.00 g, 10.98 mmol) in 6 mL of deionized water. The pH of the solution was adjusted to 2.89 using 0.1 M HCl and stirred overnight at 100 °C. The temperature was then decreased to room temperature, and water was removed using a rotary evaporator to afford a hygroscopic compound. Next, the product was washed with absolute ethanol under vigorous stirring for 4 h at room temperature. The solvent was then removed under vacuo to leave a white solid (**SI-3**). The synthesis route of **SI-3** is presented in Figure 2.

1,2-Dihydroxypropyl phosphonic acid (**SI-3**): Yield: 76%. IR ν_max_ (cm^–1^): 3249 (OH), 1041 (PO_3_). ^1^H NMR (D_2_O, 400 MHz) δ ppm: 3.99–3.92 (m, 1H, −C**H**(OH)–CH_3_), 3.46–3.42 (dd, 1H, −C**H**(OH)–PO_3_H_2_), 1.22–1.20 (d, 3H, −CH–C**H**_3_). ^31^P NMR (D_2_O, 162.00 MHz) δ ppm: 17.33.

### Static Bottle Test Protocol

The scale inhibition performance of all tested SIs against simplified calcium carbonate and calcium sulfate oilfield scales was measured according to the NACE Standard TM0374-2007 protocol.^21^ The gypsum and calcite brines were prepared, as presented in Tables 1 and 2, respectively. Furthermore, these chemicals were also screened against the Heidrun calcite scaling system, Norway. The water compositions of Heidrun brines (1:1 volume mixture of formation water and seawater) are given in Table 3. A 1000 ppm stock solution of proposed SIs was prepared in 500 mL of distilled water. In addition, the pH of the mixture solution was adjusted in the range of 4.0–6.0 to stimulate the well reservoir pH. All static bottle tests were screened in triplicates to confirm the reproducibility of the results. It was found that the standard deviation (SD) of all tests was in the range of 1–3%. Details of the scale inhibition procedure and the calculation of the calcium inhibition rate are shown in the Supporting Information.^21,25^

**Table 1:** Water Chemical Composition for the Gypsum Scale Using the NACE Standard TM0374-2007 Procedure

ion	ppm	chemical	brine 1 (g/L)^a^	brine 2 (g/L)^b^
Na^+^	5900	NaCl	7.500	7.500
Ca^2+^	3028	CaCl_2_·2H_2_O	11.100	0
SO_4_^2–^	7209	Na_2_SO_4_	0	10.660

a pH of brine 1 is 5.5.

b pH of brine 2 is 5.5.

**Table 2:** Water Chemical Composition for the Standard Calcite Scale Using the NACE Standard TM0374-2007 Procedure

ion	ppm	chemical	brine 1 (g/L)^a^	brine 2 (g/L)^b^
Na^+^	25,964	NaCl	33.000	33.000
Ca^2+^	3314	CaCl_2_·2H_2_O	12.150	0
Mg^2+^	440	MgCl_2_·6H_2_O	3.680	0
HCO_3_^–^	5346	NaHCO_3_	0	7.360

a pH of brine 1 is 5.5.

b pH of brine 2 is 7.1.

**Table 3:** Water Chemical Composition of the Heidrun Calcite Oilfield Scale (1:1 Volume Mixture of Formation Water and Seawater)

ion	ppm	component	brine 1 (g/L)^a^	brine 2 (g/L)^b^
Na^+^	39,020	NaCl	49.590	49.590
Ca^2+^	2040	CaCl_2_·2H_2_O	7.480	
Mg^2+^	530	MgCl_2_·6H_2_O	4.430	
K^+^	1090	KCl	2.078	
Ba^2+^	570	BaCl_2_·2H_2_O	1.014	
Sr^2+^	290	SrCl_2_·6H_2_O	0.882	
HCO_3_^–^	1000	NaHCO_3_	0	2.760

a pH of brine 1 is 5.5.

b pH of brine 2 is 7.1.

### Calcium Compatibility Test

Organophosphorous compounds are widely used as SIs for squeeze treatment applications.^20^ However, most of these chemicals are not compatible with producing water, including divalent cations such as Ca^2+^ ions, affording a Ca^2+^-SI precipitate. Therefore, there is a clear need to check the tolerance activity between the proposed SIs and calcium ions. A series of compatibility tests containing different concentrations of SIs (100, 1000, 10,000, and 50,000 ppm) and various Ca^2+^ ions concentrations (100, 1000, and 10,000 ppm) in the presence of 30,000 ppm NaCl (3 wt %) were dissolved in 20 mL of distilled water. The pH of the mixture solutions was adjusted in the range of 4–6. Then, all prepared vessels were located in an oven at 80 °C over 24 h. The haziness and/or precipitation of tested SIs with Ca^2+^ ions were checked at mixing, after 30 min and 1, 4, and 24 h via visual observation.

## Results and Discussion

### Chemistry

Three aminomethylene-free phosphonate-based SIs were studied as antiscaling agents for calcite and gypsum scales. First, the commercial and natural product antibiotic fosfomycin (**SI-1**) was tested as the SI under oilfield conditions. Second, its orally bioavailable derivative, fosfomycin trometamol (**SI-2**), was synthesized by the reaction of fosfomycin disodium salt (**SI-1**) and a tromethamine acid salt with oxalic acid in the presence of alcoholic media.^22^ Finally, due to the instability of **SI-1** in acidic media, it was desired to hydrolyze the epoxide ring to afford an aliphatic compound and compare its performances as new aminomethylene-free phosphonate SI (**SI-3**). This reaction consisted of bringing the pH of a fosfomycin solution down to 2.89 under reflux conditions at high temperature.^26^

The structures of these compounds were characterized using NMR and FTIR spectroscopic techniques. In the FTIR spectra of **SI-1**, a sharp absorption peak is shown at 1089 cm^–1^, attributed to the phosphonate group. As expected, there were no easily distinguishable IR bands for the CO bond of the epoxide. Furthermore, this structure can be further confirmed by the absence of an OH band (3200–3700 cm^–1^) and a C=O band (1650–1800 cm^–1^). The FTIR spectra of **SI-2** showed a remarkable broad peak at 3048 cm^–1^, representing the NH stretching vibration, and further at 2945 and 2822 cm^–1^ as a result of the OH stretch. A characteristic peak for the phosphonate group was presented at 1035 cm^–1^. In the case of **SI-3**, a broad peak for the OH stretch was obtained at 3249 cm^–1^, and a sharp absorption peak at 1041 cm^–1^ was attributed to the phosphonate group.

The ^1^H NMR for **SI-1** in D_2_O displayed multiple peaks in the range of δ 3.20–3.13 ppm, representing the −C**H**–CH_3_ proton. A doublet–doublet peak was observed at δ 2.75–2.69 ppm, attributed to the −C**H**–PO_3_H_2_ proton. Finally, a very sharp doublet peak was displayed at δ 1.38 and δ 1.37 ppm representing the methyl group (−CH–C**H**_3_). For **SI-2**, a sharp singlet peak was shown at δ 3.60 ppm representing the methylene groups (C**H**_2_(OH))_2_–C(NH_2_)–C**H**_2_−). Furthermore, the determining factor in differentiating **SI-3** from **SI-1** was the chemical shift obtained in the ^31^P NMR spectra. While **SI-1** showed a singlet signal at δ 10.00 ppm, **SI-2** and **SI-3** shifted the signal to δ 12.29 and δ 17.33 ppm, respectively.

### Static Scale Inhibition Performance

The scale inhibition performance of aminomethylene-free phosphonates (**SI-1**, **SI-2**, and **SI-3**) was screened against gypsum and calcite scales using the NACE Standard TM0374-2007 protocol.^21,23−25^ A series of numerous concentrations of SIs of 100, 50, 20, 10, 5, 2, and 1 ppm were evaluated using static bottle tests at 80 °C for 5 h. In addition, a commercial SI **HPAA** was also tested under the same conditions to compare the performance of our proposed SIs with a commercially available product that possesses a similar chemical structure.

### Gypsum Scale

**Figure 3:**
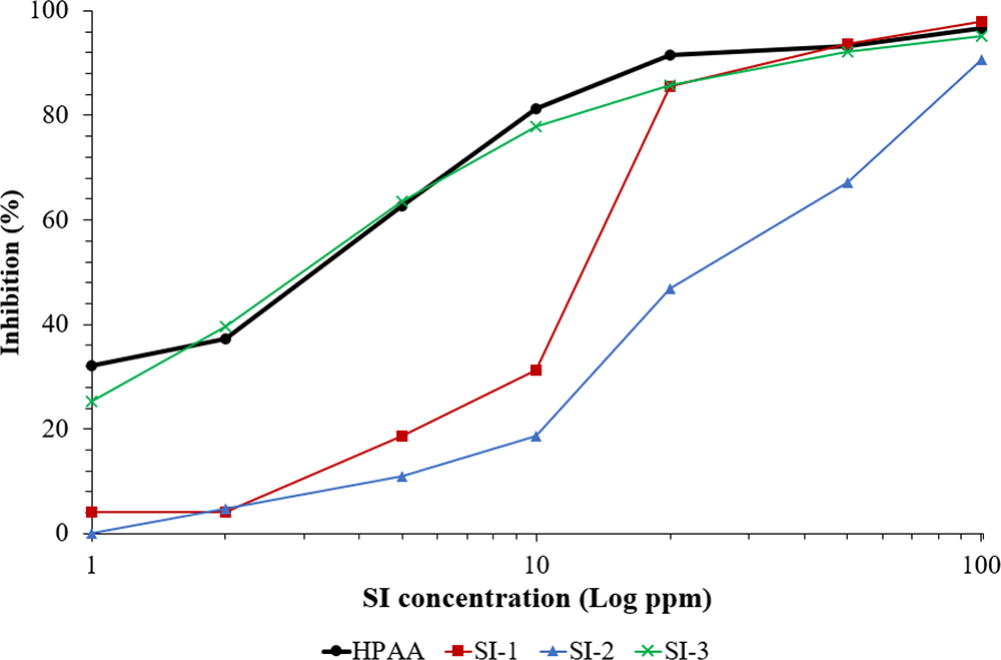
Schematic diagram of gypsum inhibition efficiency of aminomethylene-free phosphonates (**SI-1**, **SI-2**, and **SI-3**) and the commercial SI (**HPAA**).

Table 4 and Figure 3 present the inhibition activity of the commercial scale inhibitor **HPAA** and three new aminomethylene-free phosphonates (**SI-1**, **SI-2**, and **SI-3**) against the gypsum scale using static bottle tests. In general, all tested aminomethylene-free phosphonate scale inhibitors showed good inhibition performance for the gypsum scale. The commercial scale inhibitor **HPAA** gave a very good inhibition efficiency at high concentration SI doses (100–20 ppm) and a moderate inhibition activity at low SI concentrations (10–1 ppm). For example, the inhibition efficiency of **HPAA** was 97% at 100 ppm, as shown in Table 4.

**Table 4:** Gypsum Inhibition Efficiency of Aminomethylene-Free Phosphonates (**SI-1**, **SI-2**, and **SI-3**) and the Commercial SI (**HPAA**)

	% Inhibition			
SI concentration (ppm)	**HPAA**	**SI-1**	**SI-2**	**SI-3**
100	97	98	91	95
50	93	94	67	92
20	91	85	47	86
10	81	31	19	78
5	63	19	11	63
2	37	4	5	40
1	32	4	0	25

For the newly tested SI-based fosfomycin analog, **SI-1** showed only about 1% better performance than **HPAA** at 100 and 50 ppm. In addition, **SI-1** afforded a reasonable inhibition efficiency of 85% at 20 ppm. However, the inhibition efficiency was dropped down to 31% at 10 ppm. It was also investigated that **SI-1** showed a weak inhibition performance at low SI concentrations compared to **HPAA**. The modified fosfomycin (**SI-2**) containing one phosphonate group and three hydroxyl moieties in its structure backbone afforded a moderate gypsum inhibition performance compared to **HPAA**. For example, the calcium inhibition activity was 91% at 100 ppm **SI-2**. It was also found that the inhibition activity of **SI-2** was slightly dropped throughout the whole test until reaching <10% inhibition at 2 and 1 ppm (Table 4).

Finally, **SI-3** provided excellent inhibition performance at high concentration SI doses, behaving similarly to the commercial SI **HPAA**, as presented in Table 4. The static scale efficiency experiments showed that an inhibition performance of 95% was reached at 100 ppm **SI-3**. Even at low concentrations, **SI-3** showed a certain inhibition of 63% at a 5 ppm SI. Moreover, **SI-3** showed 25% inhibition at 1 ppm, while **HPAA** was slightly higher at 32% inhibition.

Generally, **HPAA** maintained the SI with the highest inhibition activity against the gypsum scale. **SI-1** and **SI-2** showed good performance only at the highest concentrations of the SI. However, **SI-3** showed good performance throughout all concentrations of SIs tested. The slightly different performance between **HPAA** and **SI-3** is attributed to the presence of the carboxyl moiety in the structure backbone of **HPAA**. The presence of this functional group in its SI structure chain allows more and stronger interactions between the SI and ions in the solution, affording a better inhibition performance.^1^

### Simplified Calcite Scale

Table 5 shows the inhibition performance of the studied SIs against the calcite scale using static bottle tests. **HPAA** presented 71 and 57% inhibition at concentrations of 100 and 50 ppm SIs, respectively, remaining above 20% until 10 ppm. **SI-1**, **SI-2**, and **SI-3** showed only about 43, 48, and 62% inhibition at 100 ppm, respectively. However, **SI-1** dropped its inhibition to 22% at 50 ppm and further to 17% at 20 ppm, while **SI-2** instantly dropped to 18% at 50 ppm. Both **SI-1** and **SI-2** presented an inhibition below 10% from 10 and 20 ppm, respectively. In the case of **SI-3**, a fair inhibition performance of 62% was obtained at 100 ppm dropping to 42 and 17% at 50 and 20 ppm, respectively.

**Table 5:** Simplified Calcite Inhibition Efficiency of Aminomethylene-Free Phosphonates (**SI-1**, **SI-2**, and **SI-3**) and the Commercial SI (**HPAA**)

	% Inhibition			
SI concentration (ppm)	**HPAA**	**SI-1**	**SI-2**	**SI-3**
100	71	43	48	62
50	57	22	18	42
20	34	17	6	17
10	29	8	1	12
5	18	6	0	9
2	3	4	0	6
1	1	1	0	1

As similarly obtained for the gypsum scale, **SI-3** improved inhibition performance compared to **SI-1**. This improvement may be because **SI-3** is a linear structure with a hydroxyl group that could provide extra binding capabilities in contrast to the epoxide ring of **SI-1**.^18^ As mentioned earlier, the chemical structure of **SI-3** is closely analogous to **HPAA** except for the carboxyl group. Due to this functional group, **HPAA** provided inhibition of more than 10% at concentrations of up to 5 ppm, which is not the case of **SI-1** or **SI-3**. Furthermore, it was expected that **SI-2** provided better performance than other tested aminomethylene-free phosphonates in this project due to the presence of the diverse amino, hydroxyl, and phosphonate groups in its structure backbone, but this was not the case.

### Heidrun Calcite Scale

In this study, the scale inhibition performance of all tested aminomethylene-free phosphonates was screened against the Heidrun calcite scale using the NACE Standard TM0374-2007 protocol. The Heidrun calcite inhibition efficiencies of aminomethylene-free phosphonates (**SI-1**, **SI-2**, and **SI-3**) and the commercial SI (**HPAA**) are presented in Table 6. The obtained results showed good inhibition performance for all tested SIs at high SI concentrations. For the commercial SI, the inhibition efficiency of **HPAA** remained above 90% until 5 ppm. In addition, it was found that the inhibition performances of **HPAA** were dropped to 36 and 13% at 2 and 1 ppm, respectively.

**Table 6:** Heidrun Calcite Inhibition Efficiency of Aminomethylene-Free Phosphonates (**SI-1**, **SI-2**, and **SI-3**) and the Commercial SI (**HPAA**)

	% Inhibition			
SI concentration (ppm)	**HPAA**	**SI-1**	**SI-2**	**SI-3**
100	100	92	68	97
50	98	71	55	73
20	97	49	34	61
10	95	38	24	42
5	95	32	18	15
2	36	24	15	10
1	13	24	13	7

For the new aminomethylene-free phosphonate SIs, fosfomycin **SI-1** (based epoxide ring) showed 92% inhibition at 100 ppm. However, this inhibition decreased to 71% at 50 ppm. Moreover, **SI-1** afforded a weak inhibition at low inhibitor doses against the Heidrun calcite scale. For the modified fosfomycin, **SI-2** showed moderate inhibition performances of 68 and 55% at 100 and 50 ppm, respectively. It was also found that the inhibition performance was dropped continuously at lower concentrations, affording poor efficiency. Furthermore, the new linear aminomethylene-free phosphonate **SI-3** showed very good inhibition efficiency against the calcite scale at high SI concentrations (100–10 ppm). For example, the inhibition efficiency of **SI-3** reached 97% at 100 ppm. Moreover, **SI-3** showed poor inhibition performances at lower inhibitor concentrations compared to other new aminomethylene-free phosphonates SIs (**SI-1** and **SI-2**) and commercial SI **HPAA**, as shown in Table 6.

Clearly, for both calcite and gypsum oilfield scales, the new aminomethylene-free phosphonate SIs are moderate inhibitors. The limited number of functional inhibition groups (e.g., PO_3_H_2_, COOH, and SO_3_H) in the inhibitor structural backbone led to weak inhibition performance, particularly at lower SI doses. In addition, the testing conditions of the Heidrun calcite water system most likely influenced the scale inhibitor performance compared to standard calcite. For standard calcite, the Ca^2+^ ions concentration in the mixed brine is 1657 ppm, and the Mg^2+^ concentration is 220 ppm. For Heidrun calcite, the Ca^2+^ concentration is 1020 ppm and the Mg^2+^ concentration is 265 ppm. Although the Mg^2+^ concentration for Heidrun conditions is slightly higher than that proposed by NACE Standard TM0374-2007, it did not seem to negatively affect the tested SIs’ inhibition performance. This may be due to the lower Ca^2+^ concentration for Heidrun water chemistry as the harmful effect of Mg^2+^ ions on the inhibition efficiency is stronger at higher Ca^2+^ concentrations.^6^

### Calcium Tolerance Tests

As mentioned earlier, most organophosphorus-based SIs showed poor compatibility activity with calcium ions, giving a Ca^2+^-SI complex, which causes several issues in oil production, e.g., formation damage.^11^ So, there is a certain need to check that the newly tested SIs will be compatible with calcium ions at various concentrations of Ca^2+^ and SIs. In addition, the tolerance test plays an essential role in squeeze scale treatment applications. In this study, we carried out a matrix of tolerance tests for all tested aminomethylene-free phosphonates (**SI-1** to **SI-3** and **HPAA**) at 80 °C. The compatibility results are presented in Tables 7–10 and Table S2). The concentrations of tested SIs were varied from 100 to 50,000 ppm, while the doses of Ca^2+^ ions were changed from 100 to 1000 ppm in the presence of 30,000 ppm NaCl to match the salinity of the petroleum reservoir.

**Table 7:** Tolerance Tests in 100 ppm Ca^2+^ and 30,000 ppm (3.0 wt %) NaCl for **HPAA**

	Appearance				
dose (ppm)	after mixing	30 mins	1 h	4 h	24 h
100	clear	clear	clear	clear	clear
1000	clear	clear	clear	clear	clear
10,000	clear	clear	clear	clear	clear
50,000	clear	clear	clear	clear	clear

**Table 8:** Tolerance Tests in 1000 ppm Ca^2+^ and 30,000 ppm (3.0 wt %) NaCl for **HPAA**

	Appearance				
dose (ppm)	after mixing	30 mins	1 h	4 h	24 h
100	clear	clear	clear	clear	clear
1000	clear	clear	clear	clear	clear
10,000	hazy	precipitate	precipitate	precipitate	precipitate
50,000	hazy	precipitate	precipitate	precipitate	precipitate

**Table 9:** Tolerance Tests in 10,000 ppm Ca^2+^ and 30,000 ppm (3.0 wt %) NaCl for **HPAA**

	Appearance				
dose (ppm)	after mixing	30 mins	1 h	4 h	24 h
100	clear	clear	clear	clear	clear
1000	hazy	precipitate	precipitate	precipitate	precipitate
10,000	hazy	precipitate	precipitate	precipitate	precipitate
50,000	hazy	precipitate	precipitate	precipitate	precipitate

**Table 10:** Tolerance Tests in 10,000 ppm Ca^2+^ and 30,000 ppm (3.0 wt %) NaCl for **SI-2**

	Appearance				
dose (ppm)	after mixing	30 mins	1 h	4 h	24 h
100	clear	clear	clear	clear	clear
1000	clear	clear	clear	clear	clear
10,000	clear	clear	clear	clear	clear
50,000	clear	clear	clear	clear	clear

**HPAA** exhibited reasonable calcium tolerance at all SI doses (100–50,000 ppm) and 100 ppm Ca^2+^ ions (Table 7). However, **HPAA** presented poor calcium compatibility at high calcium ion concentrations. For example, 10,000 ppm **HPAA** gave haziness and precipitates with 1000 and 10,000 ppm Ca^2+^ ions over a 24 h test period, as shown in Tables 8 and 9, respectively.

The newly tested aminomethylene-free phosphonates (**SI-1** to **SI-3**) displayed outstanding calcium compatibility. No precipitates were determined at all inhibitor and calcium ion concentrations. For example, **SI-1** and **SI-2** gave superior calcium compatibility performance at all SI concentrations and 10,000 ppm calcium ions (Table 10 and Table S2). The presence of ether linkages in their structure backbones may enhance the ability to overcome complexing with Ca^2+^ ions, as investigated previously in our research.^20^ It was also found that **SI-3** (close analog of **HPAA**) displayed cloudiness at 10,000, and 50,000 ppm inhibitors and 10,000 ppm Ca^2+^ ions, as shown in Table S2.

## Conclusions

Three new aminomethylene-free phosphonate-based nontoxic fosfomycin drugs have been developed as scale inhibitors in the petroleum industry. The new aminomethylene-free phosphonates (namely, fosfomycin disodium salt **SI-1**, fosfomycin trometamol **SI-2**, and 1,2-dihydroxypropyl phosphonic acid **SI-3**) have been compared and screened with commercial SI hydroxyphosphonoacetic acid (**HPAA**) through a static bottle test and calcium compatibility. For the gypsum scale, **SI-1** and **SI-2** showed good performance only at the highest concentrations of the SI. However, **SI-3** showed good performance throughout all concentrations of SIs tested. In addition, **HPAA** exhibited very good scale inhibition efficiency. The slightly different performance between **HPAA** and **SI-3** is attributed to the presence of the carboxyl group in the structure of **HPAA**. For the calcite scale, the inhibition efficiency of all tested SIs was relatively moderate for simplified calcium carbonate, according to the NACE Standard TM0374-2007 protocol. However, these chemicals gave good inhibition activity against the Heidrun calcite scale, Norway. We speculate that the primary reason for the weak inhibition of these SI classes may be due to the limited number of functional inhibition groups (e.g., PO_3_H_2_, COOH, and SO_3_H) in the inhibitor structural backbone.

Commerical inhibitor **HPAA** gave poor compatibility with Ca^2+^ up to 10,000 ppm. Interestingly, the new aminomethylene-free phosphonates (**SI-1** and **SI-2**) gave superior compatibility activity at all inhibitor connections (up to 50,000 ppm) and high calcium ion doses up to 10,000 ppm. We believe that the ether linkage in their structure backbones improved the calcium tolerance properties. Moreover, **SI-3** (close analog of **HPAA**) displayed outstanding calcium tolerance up to 1000 ppm calcium ions. However, **SI-3** afforded haziness at 10,000 and 50,000 ppm inhibitors and 10,000 ppm Ca^2+^ ions. We plan to study the thermal stability and adsorption/desorption activities of these nontoxic phosphonate scale inhibitors for squeeze treatment applications.

## Supporting Information Available

Evaluation of the scale inhibition performances, Table S1: dosed solutions for static performance tests of SIs, Table S2: Ca^2+^ tolerance tests at 30,000 ppm (3 wt %) NaCl for **SI-1** to **SI-3**, Figure S1: duration test results for static bottle tests, and ^1^H and ^31^P NMR spectra of **SI-1**, **SI-2**, and **SI-3 (Figures 2-4)** (PDF)
