# Supplementary material for: Fosfomycin and Its Derivatives: New Scale Inhibitors for Oilfield Applications
Source: ACS Omega. 2022 Mar 15;7(12):10701–8. doi: 10.1021/acsomega.2c00429 (PMC8973091; doi:10.1021/acsomega.2c00429)
Supplement: Supplementary file 1 — ao2c00429_si_001.pdf [file ao2c00429_si_001.pdf]

Supporting Information for:

Fosfomycin and Its Derivatives: New Scale Inhibitors for Oilfield Applications

Mohamed F. Mady[a,b*] and Rocio Ortega[a],

[a]Department of Chemistry, Bioscience and Environmental Engineering, Faculty of Science and Technology, University of Stavanger, N-4036 Stavanger, Norway.
[b]Department of Green Chemistry, National Research Centre, Dokki, Cairo 12622, Egypt.

Contents:

Evaluation of the scale inhibition performances	S2-S4
**Figure S1**. Duration test results for static bottle tests.	S3
**Figure S2**. ^{1}H and ^{31}P NMR spectra of **SI-1.**	S6
**Figure S3**. ^{1}H and ^{31}P NMR spectra of **SI-2.**	S7
**Figure S4**. ^{1}H and ^{31}P NMR spectra of **SI-3.**	S8
**Table S1.** Dosed solutions for static performance tests of SIs.	S2
**Table S2.** Ca^{2+} tolerance tests at 30000 ppm (3 wt%) NaCl for **SI-1** to **SI-3**.	S5

Evaluation of the Scale Inhibition Performances

Laboratory static test protocol was carried out for all new synthesized SIs for calcium carbonate (calcite) and calcium sulphate (gypsum) oilfield scales. The static antiscaling measurement was determined according to the NACE Standard TM0374-2007 protocol. For gypsum and calcite scales, the brines were prepared as described in Tables 1 and 2 in the manuscript, respectively. We also screened these chemicals against the calcite scale according to the Heidrun oilfield, Norwegian Sea, Norway. The compositions of Heidrun brines (50:50 mix of formation water and seawater) are given in Table 3 in the manuscript. Additionally, a 1000 ppm stock solution of SIs was prepared in 500 mL of deionized water, and the pH was adjusted between 4 to 6 to resemble a typical produced water from a petroleum reservoir.

Cationic and anionic brines (B1 and B2, respectively) were prepared accordingly, as described in Tables 1, 2, and 3 in the manuscript. A total volume of 40 mL of 1:1 solution of cationic brine (B1) and anionic brine (B2) was used to produce the corresponding scale, e.g., calcite and gypsum. Consequently, in 50 mL Schott Duran® glass bottles, different concentrations of SI were dosed into known volumes of B1 and B2 by diluting a 1000 ppm stock solution of SI. Details of the procedure are shown in Table S1. Automated pipettes of 10 mL, 100, and 1000 µL (Thermo Fisher Scientific, USA) were used for this purpose. A series of SI concentrations are set to 100, 50, 20, 10, 5, 2, and 1 ppm (Table S1). Two blank samples were also prepared.

Table S1. Dosed solutions for static performance tests of SIs.

SI concentration (ppm)	B1 (mL)	B2 (mL)	1000 ppm stock SI (mL)
100	20	16	4
50	20	18	2
20	20	19.2	0.8
10	20	19.6	0.4
5	20	19.8	0.2
2	20	19.92	0.08
1	20	19.96	0.04
0	20	20	0

To determine the scaling time that samples required to be spent in the oven, a series of jar tests containing blank samples were performed prior to SI testing. The blank samples were placed in an oven at 80 °C for 1, 2, 3, 4, 5, 6, and 24 hours. These samples were then analyzed to determine the Ca^{2+} concentration in the solution via titration with EDTA using an ammonium purpurate (also known as murexide) indicator. These experiments were evaluated in triplicates to confirm the reproducibility of the obtained results. The standard deviation of all experiments was in the range of 1−5%. Unfortunately, the main drawback of this static jar test is the range variation of the obtained standard deviation. It was found that the ideal scaling time for forming the maximum amount of oilfield scale is 5 hours over the test period (1-24 h), as shown in Figure S1. After this time, no significant change in the Ca^{2+} concentration retained in the solution was detected.

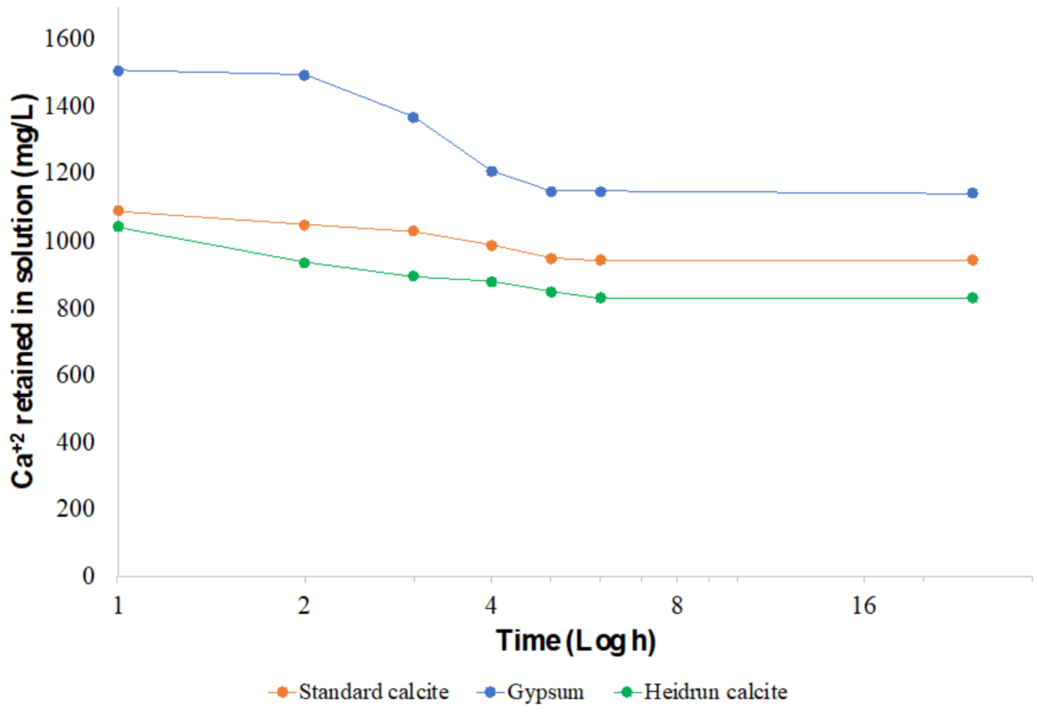

Figure S1. Duration test results for static bottle tests.

All samples containing SIs were prepared, mixed thoroughly, capped tightly, and then placed in a pre-heated oven at 80ºC and kept for 5 hours. The Ca^{2+} concentration in each sample was

determined following the procedure given by ASTM D-511. The analysis consisted of withdrawing a 1 mL aliquot (without prior filtration) and diluting it to 50 mL with deionized water in a conic flask. Consequently, pH was adjusted to 12-13 with 100 μL of NaOH 50% and 200 μL of murexide indicator were added. The titration setup consisted of a 25:0.05 mL glass burette (Hirschmann™, Germany) and a Lab Disc (VWR®, Germany) stirrer. The sample was then titrated with EDTA 0.01 M until a color change from pink to purple occurred. The volume of EDTA consumed was recorded, and the Ca^{2+} concentration was estimated as shown in Equation 1;

$$Ca^{2+}(ppm) = \frac{A \times B}{D} \times 40100 \quad (1)$$

where A is the EDTA volume required to titrate Ca^{2+} in the sample (mL), B is the concentration of EDTA (mol/L), D is the volume of aliquot used (1 mL), and 40100 represents the molecular weight of Ca^{2+} in mg/mol.

The scale inhibition efficiency is calculated based on the concentration of Ca^{2+} retained in solution relative to the blank at room temperature. The percent inhibition values were calculated as follows;

$$\%\ inhibition = \frac{C_a - C_b}{C_o - C_b} \times 100 \quad (2)$$

where C_a refers to the Ca^{2+} concentration in the sample after 5 hours, C_b is the Ca^{2+} concentration in the blank after 5 hours, and C_o is the Ca^{2+} concentration in the blank before precipitation. Each sample was titrated in triplicates.

Table S2. Ca^{2+} tolerance tests at 30000 ppm (3 wt%) NaCl for **SI-1** to **SI-3**.

SI	Ca^{2+} dose (ppm)	SI dose (ppm)	Appearance				
			after mixing	30 minutes	1 hour	4 hours	24 hours
**SI-1**	1000	100	clear	clear	clear	clear	clear
		1000	clear	clear	clear	clear	clear
		10000	clear	clear	clear	clear	clear
		50000	clear	clear	clear	clear	clear
	10000	100	clear	clear	clear	clear	clear
		1000	clear	clear	clear	clear	clear
		10000	clear	clear	clear	clear	clear
		50000	clear	clear	clear	clear	clear
**SI-2**	1000	100	clear	clear	clear	clear	clear
		1000	clear	clear	clear	clear	clear
		10000	clear	clear	clear	clear	clear
		50000	clear	clear	clear	clear	clear
	10000	100	clear	clear	clear	clear	clear
		1000	clear	clear	clear	clear	clear
		10000	clear	clear	clear	clear	clear
		50000	clear	clear	clear	clear	clear
**SI-3**	100	100	clear	clear	clear	clear	clear
		1000	clear	clear	clear	clear	clear
		10000	clear	clear	clear	clear	clear
		50000	clear	clear	clear	clear	clear
	1000	100	clear	clear	clear	clear	clear
		1000	clear	clear	clear	clear	clear
		10000	clear	clear	clear	clear	clear
		50000	clear	clear	clear	clear	clear
	10000	100	clear	clear	clear	clear	clear
		1000	clear	clear	clear	clear	clear
		10000	clear	hazy	hazy	hazy	hazy
		50000	hazy	hazy	hazy	hazy	hazy

➢ ^{1}H, and ^{31}P NMR spectra of SI-1, SI-2, and SI-3

S1-1 ^{1}H NMR

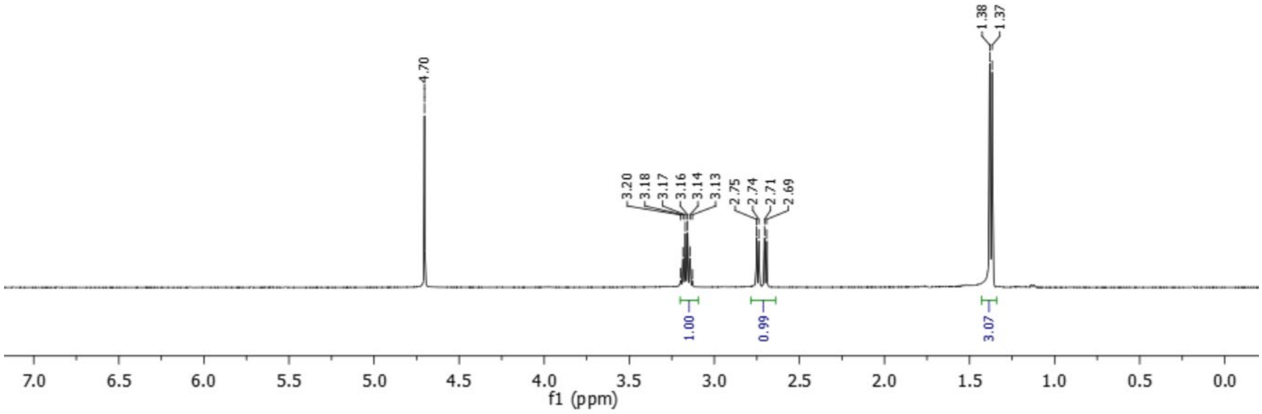

S1-1 ^{31}P NMR

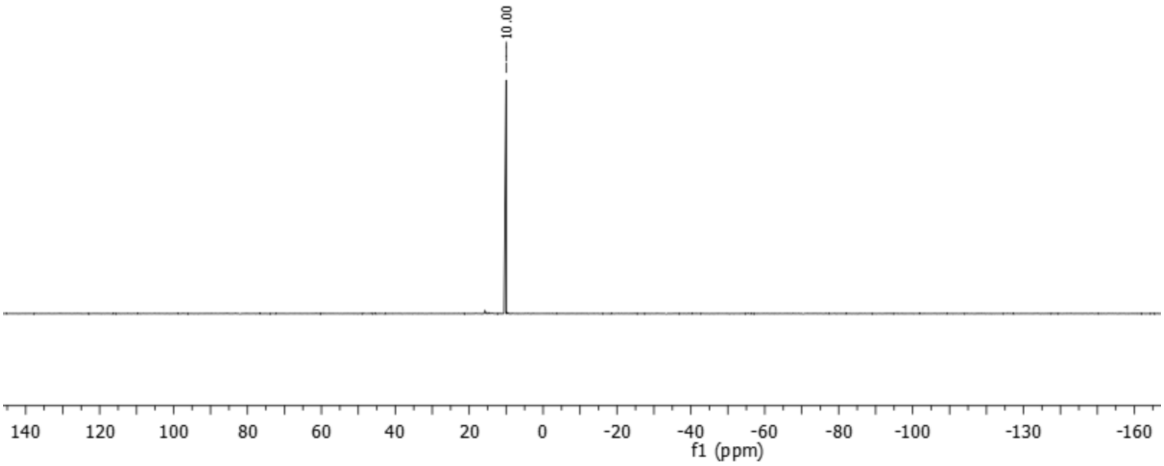

Figure S2. ^{1}H and ^{31}P NMR spectra of SI-1

SI-2 ^{1}H NMR

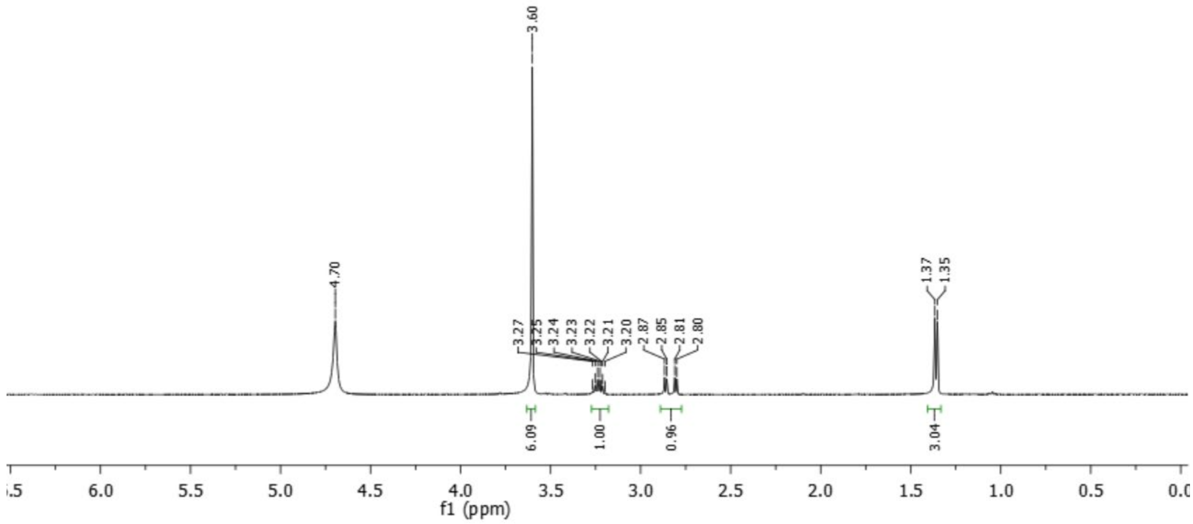

S1-2 ^{31}P-NMR

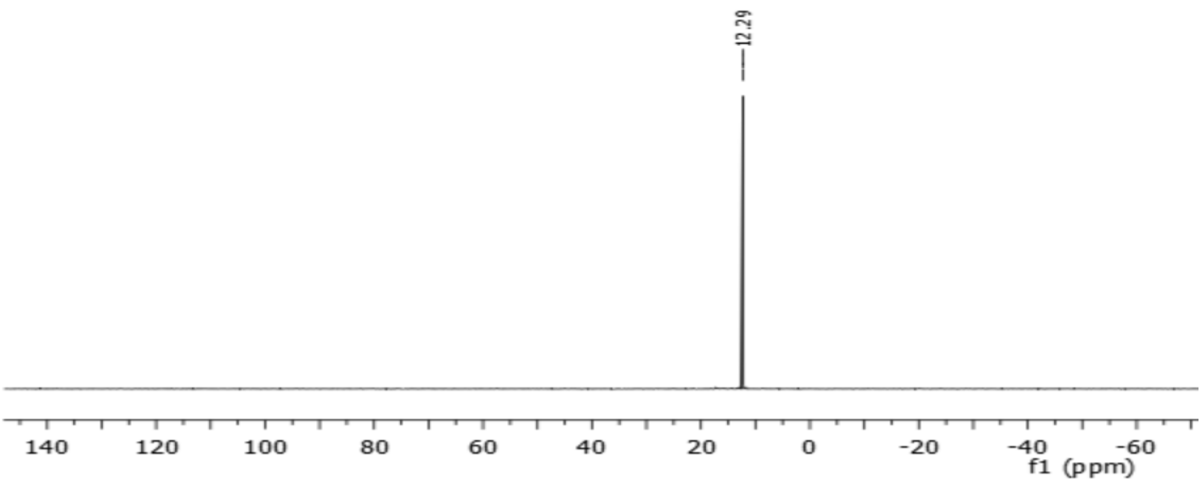

Figure S3. ^{1}H and ^{31}P NMR spectra of SI-2

SI-3 ^{1}HNMR

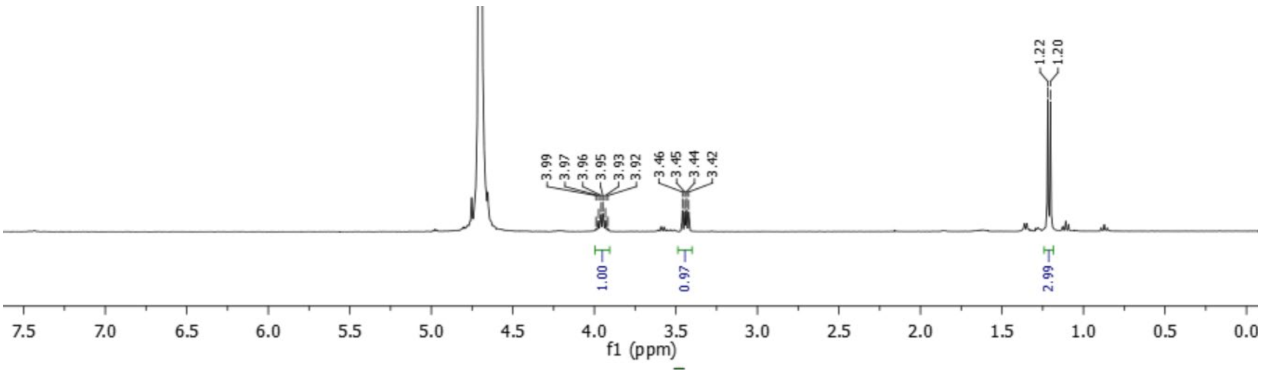

SI-3 ^{31}P NMR

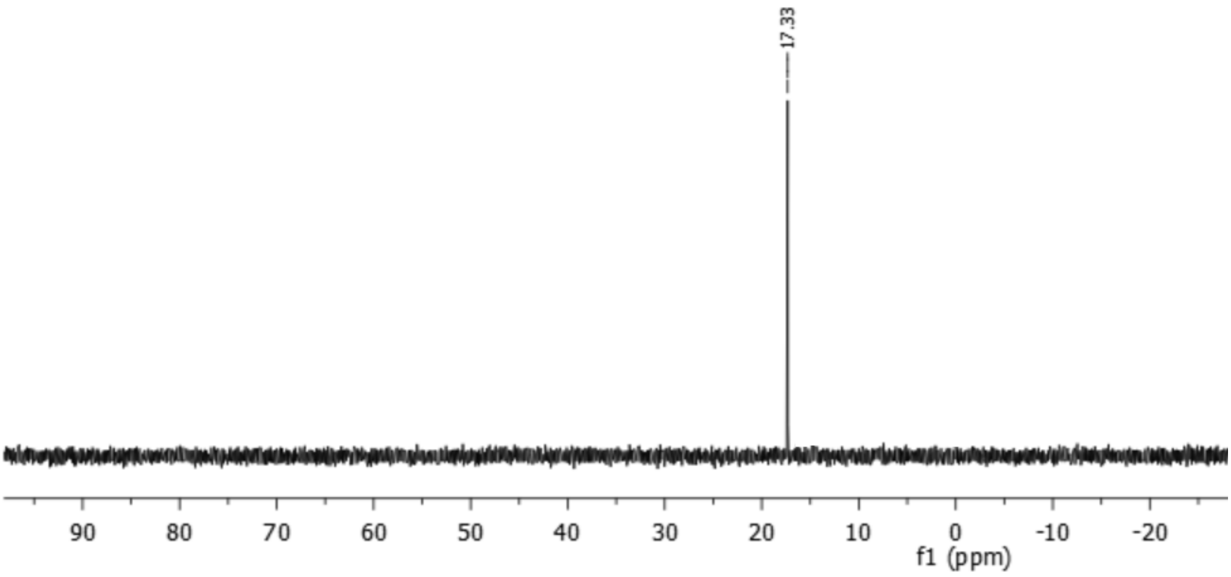

Figure S4: ^{1}H and ^{31}P NMR spectra of SI-3.
